# Challenges in Diagnosing Sudden Death Caused by Acute Hemorrhagic Pancreatitis: An Autopsy-Based Case Report

**DOI:** 10.7759/cureus.49500

**Published:** 2023-11-27

**Authors:** Lakhan Lal Navlani, Arushi Verma, Raviprakash Meshram, Vikas Vaibhav, Shailesh V Parate

**Affiliations:** 1 Forensic Medicine and Toxicology, All India Institute of Medical Sciences, Rishikesh, IND

**Keywords:** pathophysiological insights, hemorrhagic, alcohol, pancreatitis, sudden death

## Abstract

Forensic pathologists frequently encounter cases of sudden natural death. Most sudden natural deaths are attributed to cardiac causes. Acute pancreatitis, especially hemorrhagic pancreatitis, is an infrequent yet critical contributor to sudden death. The role of a forensic pathologist is imperative in such cases to find out the cause of the sudden death and to either confirm or refute any allegations. In this context, we describe a case of a 34-year-old male who experienced sudden death due to acute hemorrhagic pancreatitis, highlighting the need for a detailed autopsy, pathophysiological insights, and diagnostic challenges.

## Introduction

The term "sudden death," also called "sudden and unexpected natural death," is defined by the WHO as "death occurring within 24 hours after the onset of the symptoms [1]." These deaths, often occurring without any prior warning symptoms or medical intervention, present a profound challenge for forensic pathologists, investigating police officers, and society as a whole. Moreover, many such cases are not necessarily "unexpected," and conversely, some unexpected deaths may not be "sudden." Therefore, it is of utmost importance to conduct postmortem examinations in these cases to unveil the cause of death and rule out any unnatural causes that can have significant implications for the investigating officers, family members of the deceased, hospital administrations, and insurance companies [2]. The predominant cause of sudden death is associated with the cardiovascular system accounting for 45% of cases. Meanwhile, 25% of cases of sudden death are linked to the respiratory system, 20% to the nervous system, and the remaining 10% are attributed to various other causes [3].

Acute pancreatitis is characterized by inflammation of the pancreas developed over a short period of time, involving parenchymal edema and necrosis resulting from auto-digestion by its own glandular enzymes, which can lead to multi-organ failure or death [4]. Acute pancreatitis is relatively uncommon, representing less than 1% of cases of sudden, unexpected deaths [5,6]. In this report, we describe a case of sudden death resulting from acute hemorrhagic pancreatitis based on autopsy findings.

## Case presentation

A case of a 34-year-old male who was found lying unresponsive by the side of the road and declared dead on arrival at the hospital was received for postmortem examination at the mortuary in the Department of Forensic Medicine and Toxicology at All India Institute of Medical Sciences, Rishikesh, India.

**Figure 1:**
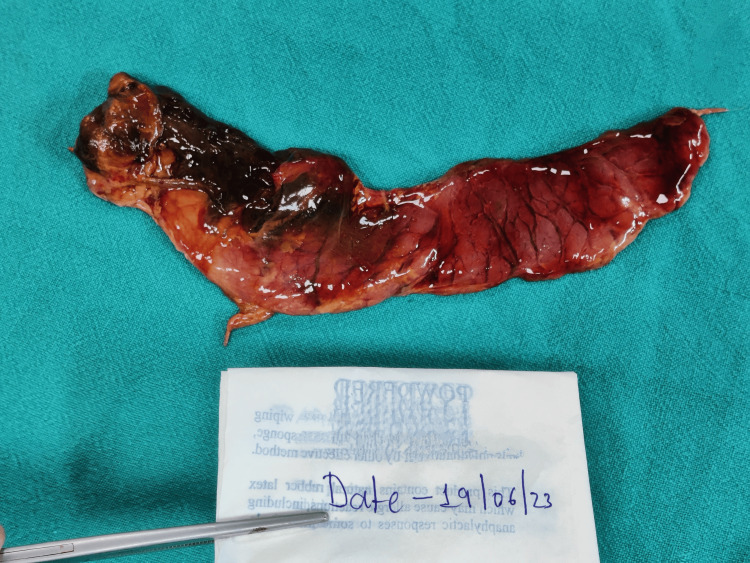
Pancreas (gross-front view) showing areas of hemorrhage at places and congestion

**Figure 2:**
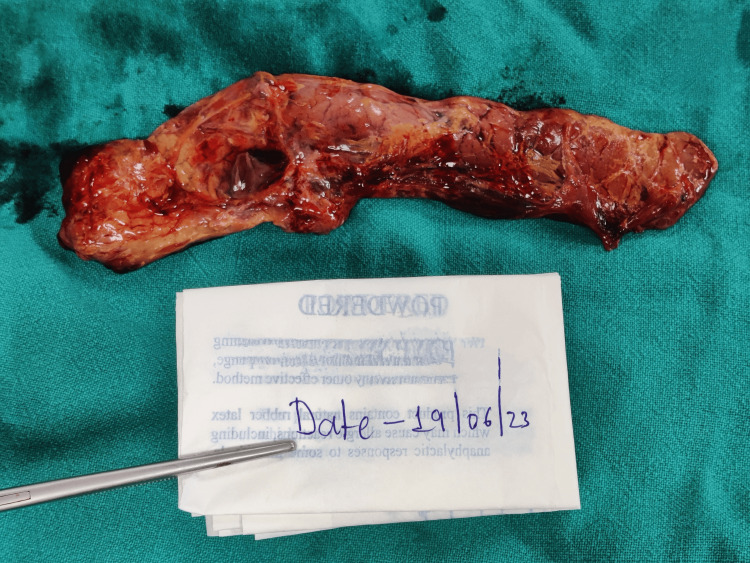
Pancreas (gross-posterior view) showing areas of hemorrhage at places and congestion

The deceased had a history of chronic alcohol consumption. There were no known co-morbidities, no history of any drug intake, no history of past surgical interventions, and no history of sudden deaths in family members. During the autopsy, on external examination, hypostasis was present over the dependent areas of the back. Rigor mortis is present in the bilateral upper and lower limbs. A greenish discoloration was present over the right iliac fossa. There were a few reddish abrasions present over the body at places. On internal examination, no injuries were found. Blood and blood clots were present in the retroperitoneal space surrounding the pancreatic region. The pancreas weighed 100 gms, was soft, congested, and had multiple reddish-brown hemorrhagic areas at places (Figures 1-2). Multiple yellowish-green stones varying in size from 1.2 cm x 0.2 cm to 0.5 cm x 0.3 cm were present in the gall bladder. The cut section of the pancreas revealed red- and yellow-colored areas present at places. The stomach contained about 20 ml of pinkish fluid having a fruity odor. Both lungs were congested and edematous (weight: right 590 gm, left 460 gm). The rest of the organs showed congestion.

**Figure 3:**
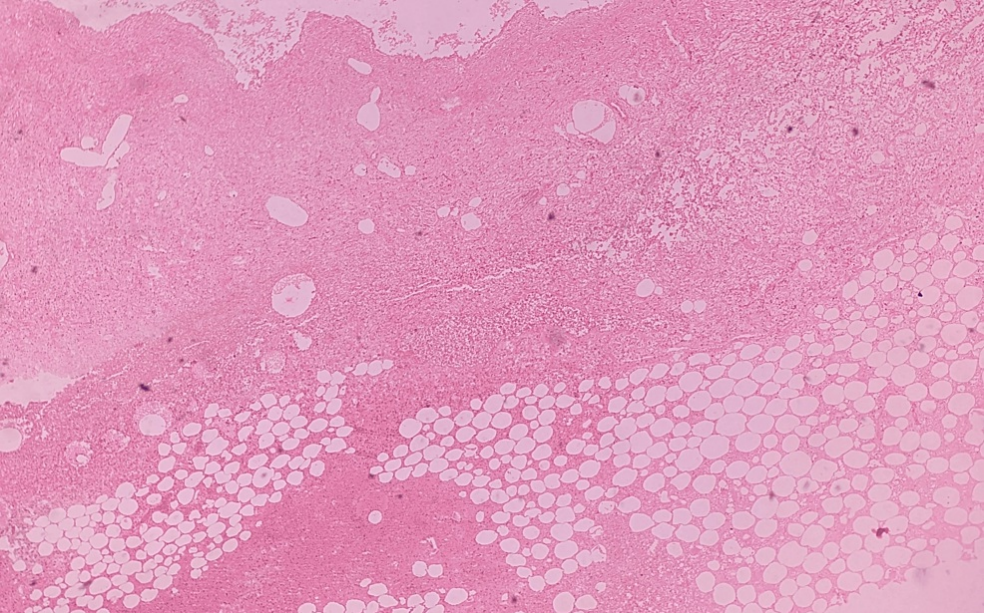
Histopathology image of the pancreas showing predominant areas of necrosis, hemorrhage, and fat necrosis along with mixed inflammatory infiltrates (H & E x 10)

**Figure 4:**
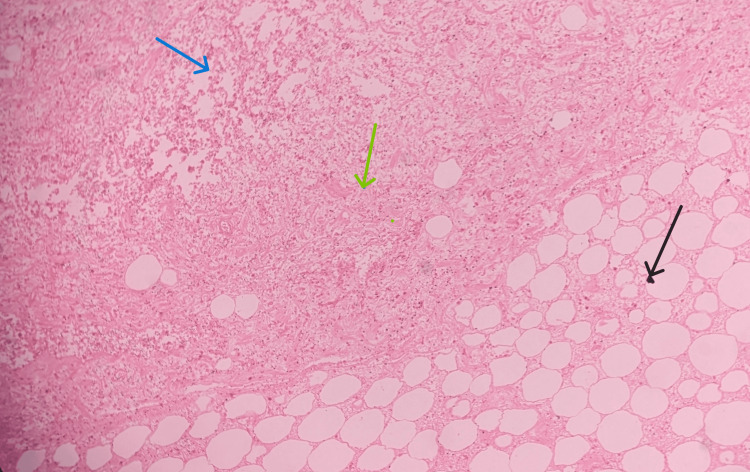
Histopathology image of the pancreas showing areas of necrosis (green arrow), areas of hemorrhage (blue arrow), and areas of fat necrosis (black arrow) along with mixed inflammatory infiltrates (H & E x 20)

After the autopsy, pieces of pancreatic tissue were retained and preserved in 10% formalin for histopathological examination. The histopathological examination of the pancreatic tissue revealed the presence of predominant areas of necrosis, hemorrhage, and fatty necrosis along with mixed inflammatory infiltrates (Figures 3-4). The cause of death was given as hemorrhagic pancreatitis and its complications. Injuries present were superficial in nature, caused by blunt force/surface impact, and were not sufficient to cause death in the ordinary course of nature.

## Discussion

Acute pancreatitis is characterized by inflammation of the pancreas, involving parenchymal edema and necrosis resulting from auto-digestion by its own glandular enzymes, which can lead to multi-organ failure or death [4]. The worldwide incidence of acute pancreatitis varies from five to 80 cases per 100,000 population [7]. The spectrum of acute pancreatitis morphology spans from mild inflammation and interstitial edema (mild acute pancreatitis) to extensive hemorrhage and necrosis (severe acute pancreatitis or hemorrhagic pancreatitis). Mild acute pancreatitis typically resolves on its own, and the overall mortality rate is less than 1%. Mild acute pancreatitis tends to be self-limiting with an overall mortality rate of less than 1%, whereas the mortality rate ranges from 10% to 30% in severe acute pancreatitis [8,9].

Although the majority of sudden death cases presenting to a forensic pathologist are due to cardiac causes, occasionally acute pancreatitis can cause sudden, unexpected deaths, making it relevant in the forensic context. The studies indicate that the occurrence of acute pancreatitis first identified during autopsy ranges from 30% to 42% [10,11]. Death can occur due to acute pancreatitis without hemorrhage; however, autopsy-based studies more frequently highlight hemorrhagic pancreatitis in cases of sudden death.

Most cases of acute pancreatitis result from either obstruction of the pancreatic duct by gallstones or chronic alcohol abuse. Together, they contribute to around 75% of all acute pancreatitis cases [12]. Pancreatitis is caused by alcohol through a set of interconnected mechanisms. It temporarily enhances the contraction of the sphincter of Oddi, resulting in the intracellular buildup of digestive enzymes, triggering their premature activation and release. Persistent alcohol consumption raises the protein content of pancreatic enzymes and contributes to the deposition of protein plugs that obstruct the outflow of the pancreatic duct. It also has direct toxic effects on acinar cells [13]. In our case study, the deceased had a history of chronic alcohol consumption, and on autopsy, multiple yellowish-green gallstones varying in size from 1.2 cm x 0.2 cm to 0.5 cm x 0.3 cm were present inside the gallbladder which is consistent with the abovementioned finding. Other causes of acute pancreatitis include metabolic disorders like hypertriglyceridemia and hypercalcemic conditions, hereditary factors, injuries resulting from trauma or ischemia to acinar cells, medications such as furosemide, azathioprine, and estrogens, among others, and a range of infections, including mumps [14].

Acute pancreatitis is linked to several life-threatening complications, both at the local and systemic levels. Local complications encompass gastrointestinal bleeding and adjacent bowel necrosis, potentially culminating in multiple organ failure. Systemic complications involve acute renal failure, acute respiratory distress syndrome, coagulopathy, shock, hypocalcemia, and hyperglycemia. Additionally, there is a risk of splenic vein thrombosis, which may result in variceal bleeding and pseudoaneurysm formation [15,16]. Notably, the incidence of life-threatening hemorrhage involving the peritoneal cavity, gastrointestinal tract, and retroperitoneum is estimated to be 1-3% in acute pancreatitis patients; however, it carries a high mortality rate of 50-80% [17]. Significant retroperitoneal hemorrhage can disrupt the posterior peritoneal lining, leading to hemorrhage in the peritoneal cavity (hemoperitoneum) [18]. Our case showed the presence of retroperitoneal hemorrhage only surrounding the pancreatic region. The external appearance of the pancreas in acute pancreatitis can vary greatly depending on the severity of the disease ranging from mild focal hyperemia to widespread hemorrhage and necrosis. Pancreatic substances may show interspersed foci of yellow-white chalky fat necrosis due to the enzymatic activity of lipase [14]. In our case, the pancreas showed multiple reddish-brown hemorrhagic areas along with a loss of normal architecture of the pancreas.

Despite the varied etiology, the pathophysiology of acute pancreatitis involves damage to acinar cells which leads to the release of digestive enzymes in the surrounding tissue which causes further injury. This process of developing acute pancreatitis is very similar to the postmortem autolysis of the pancreas, wherein disruption of the cell membrane leads to the release of digestive enzymes contained in the cells, causing degeneration and necrosis of the pancreas. Therefore, it is imperative for a forensic pathologist to differentiate genuine cases of sudden death due to acute pancreatitis from such postmortem artifacts. This can be best confirmed by microscopic examination of the pancreatic tissue, which reveals the presence of inflammatory infiltrates. When diagnosing acute pancreatitis, it's essential to consider additional factors like the body's state of decomposition, body position, and the distribution of changes in the pancreas, as postmortem autolysis of the pancreas is often linked to advanced decomposition, a prone body position, and widespread interstitial hyperemia/hemorrhage [14]. In our case, a histopathological examination of the pancreas revealed the presence of inflammatory infiltrates, which confirmed findings in the pancreas to be antemortem. In the study conducted by Shetty et al. [19], seven cases were examined, and in all the cases, the pancreas was edematous with hemorrhagic infiltrate. However, in our case, gross and histopathological examination of the pancreas did not reveal the presence of edema. In all seven cases, the organs showed congestion, and the brain and lungs were found to be edematous. These findings are consistent with the findings of our study, except that the brain was normal in our case.

Other ancillary investigations that can assist in confirming the postmortem diagnosis of acute pancreatitis include the analysis of vitreous humor and serum amylase and lipase. This analysis can show increased glucose levels exceeding 200 mcg/dL, indicative of hyperglycemia, and increased concentrations of vitreous urea nitrogen and creatinine, suggestive of renal failure. Postmortem serum analysis of amylase and lipase may also prove to be valuable, but it's important to note that the results of these analyses can be influenced by the postmortem interval [20]. Furthermore, with the increasing utilization of postmortem imaging studies like CT scans in the field of forensic medicine, the diagnosis of acute pancreatitis can also be supported through these examinations.

## Conclusions

Forensic pathologists often encounter cases of sudden, unexpected deaths that require a medico-legal autopsy to determine the cause of death. While cardiac causes are common, acute pancreatitis, linked to cholelithiasis and alcohol abuse, can also cause sudden death in some cases. In our case, the cause of death was given as hemorrhagic pancreatitis and its complications, highlighting the significance of hemorrhagic pancreatitis as a significant contributor to sudden deaths. However, identifying acute pancreatitis solely on the basis of gross autopsy findings can be challenging, particularly in cases where decomposition changes have set in. This case report highlights the significance of conducting a histopathological examination of the pancreas in all such cases to aid in confirming whether the changes in the pancreas are antemortem or post-mortem and consequently help in providing a precise cause of death after post-mortem examination.
